# Clear Cell Carcinoma Arising from Cesarean Section Scar Endometriosis: Case Report and Review of the Literature

**DOI:** 10.1155/2014/642483

**Published:** 2014-11-02

**Authors:** Sakura Ijichi, Taisuke Mori, Izumi Suganuma, Takuro Yamamoto, Hiroshi Matsushima, Fumitake Ito, Makoto Akiyama, Izumi Kusuki, Jo Kitawaki

**Affiliations:** Department of Obstetrics and Gynecology, Graduate School of Medical Science, Kyoto Prefectural University of Medicine, 465 Kajii-cho, Kawaramachi-Hirokoji, Kamigyo-ku, Kyoto 602-8566, Japan

## Abstract

*Introduction.* The incidence of endometriosis affecting skin tissue represents only 0.5–1.0% of all endometriosis cases. A malignancy in the abdominal wall arising from endometriosis following cesarean section is even rarer; only 21 cases have previously been reported. The therapeutic strategy has not been determined because of the limited cases. We report a case of clear cell adenocarcinoma arising in the abdominal wall from endometriosis tissues following cesarean section and review previous literature to achieve the optimal treatment and better prognosis. *Case Presentation.* A 60-year-old woman presented with a growing mass at the left side of a cesarean section scar. Radical resection of the abdominal wall mass was performed. Histopathological examination showed a clear cell adenocarcinoma. Benign endometrium-like tissues were found adjacent to the cancer lesion in the excised specimen, suggesting malignant transformation from endometriosis of the abdominal wall. *Discussion.* Local resection was performed in 10 cases (47.6%) and total abdominal hysterectomy or oophorectomy was conducted in 11 cases (52.4%). No malignant lesions were observed in either the uterus or adnexa that were resected. These cases may be expected to increase with increasing incidence of cesarean section. The significance of the extensional resection should be further elucidated.

## 1. Introduction

Endometriosis is a common disease that occurs in 5–10% of women of reproductive age, typically affecting the pelvic organs. Extrapelvic endometriosis is an uncommon event, known as “deep infiltrating endometriosis (DIE).” Endometriosis in the abdominal wall involves scar tissue resulting from gynecological procedures [1]. Overall, malignant transformation of DIE of any type is rare. Here, we report a case of clear cell carcinoma arising in the abdominal wall from endometriosis tissue following cesarean section.

## 2. Case Presentation

A 60-year-old woman, gravida 3 para 2, presented with a growing mass at the left side of a cesarean section scar (lower abdominal longitudinal incision). She had no relevant medical history and had undergone cesarean section twice, the first in 1977 due to breech presentation and again in 1979. She had no pertinent family history other than breast cancer diagnosed in her sister and had experienced menopause at 50 years of age.

**Figure 1:**
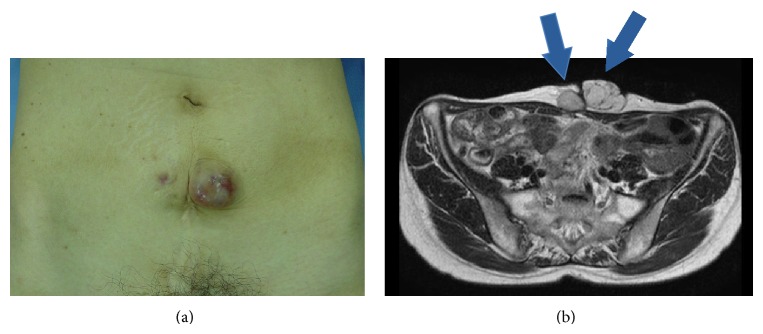
(a) Smooth mass on the middle-left side of the cesarean median scar. (b) Pelvic MRI (T2 weighted image, axial section). MRI shows the tumor associated with cesarean section scar. The right side of the scar consists of solid components and the tumor at the left side of the scar is polycystic.

**Figure 2:**
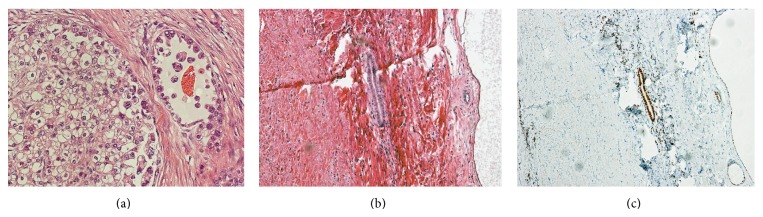
(a) In pathologic examination, many hobnail-shaped cells and clear cell were found. These are characteristic of clear cell adenocarcinoma (hematoxylin and eosin stain, magnification ×100). (b) Pathologic examination showed clear cell adenocarcinoma and endometriosis. It suggests a malignant transformation from endometriosis of the abdominal wall (hematoxylin and eosin stain, magnification ×100). (c) Staining estrogen receptor (ER), magnification ×100.

The patient noticed the nodule near the abdominal operation scar with no tenderness 4 years before presentation. The nodule grew quickly in size with no significant pain, even during menstruation. Physical examination revealed a smooth mass measuring 4 cm in diameter on the middle-left side of the cesarean median scar (Figure 1(a)). A biopsy of the mass showed atypical cells, and subsequent pelvic magnetic resonance imaging (MRI) showed two lesions, measuring 2.5 × 3.3 cm and 3.3 × 4.0 cm along the abdominal scar (Figure 2(b)). The mass located at the right side of the scar consisted of solid components, while that on the left was polycystic. There were no obvious mass-like lesions in the intraperitoneal cavity or any of the abdominal or pelvic lymph nodes. Laboratory tests revealed no increase in the serum levels of tumor markers (CEA, CA19-9, and CA125). Radical resection of the abdominal wall mass was performed with adequate margins under general anesthesia. Histopathological examination showed clear cell adenocarcinoma (Figure 2(a)), suggesting malignant transformation from endometriosis of the abdominal wall. Positron emission tomography (PET) showed no evidence of malignancy, including in the uterus, bilateral ovaries, and pelvic lymph nodes. Considering these findings together, we diagnosed clear cell adenocarcinoma of the abdominal wall arising from endometriosis after cesarean section. Eight months after the resection, a nodular lesion appeared in the patient's abdominal scar again. MRI and PET scan showed local recurrence, and she was hospitalized for resection of the recurring tumor and abdominal wall reconstruction. Histopathological examination showed the lesion to be clear cell adenocarcinoma. At 15 months after the second operation, there was no further evidence of the disease on imaging studies or clinical examination.

## 3. Discussion

Endometriosis in the extrapelvic organs is rather rare. Moreover, endometriosis affecting skin tissue is even rarer; its incidence represents only 0.5–1.0% of all endometriosis cases, being typically found at the site of surgical scars. In this case, we discovered a malignancy in the abdominal wall arising from endometriosis following cesarean section. Previous reports concerning malignant transformation of abdominal wall endometriosis are very few (only 21 cases found in a literature review; we used the following keywords: clear cell adenocarcinoma, endometriosis, and malignant transformation); the details from these reports are shown in Table 1. Among these, in 19 cases (90.5%), malignancy occurred at the site of the cesarean section scar. The remaining two cases resulted from a scar of gynecological surgery involving myomectomy and hysterectomy, suggesting that the interfusion of endometrium into the abdominal wall at the time of surgery may contribute to carcinogenesis.

The interfusion of endometrium into the abdominal wall affects 1% of women undergoing intrapelvic surgery [2], indicating that scar endometriosis is caused by iatrogenic factors [3]. Malignant transformation of endometriosis occurs in 0.75% of women who suffer from endometriosis, and in nearly 20% of cases, it occurs at extraovarian sites [4]. As shown in Table 1, the histological characteristics of malignant transformation in endometriosis of the abdominal wall are primarily represented by clear cell carcinoma (15/21; 71.4%), followed by endometrioid adenocarcinoma (3/21; 14.3%), serous adenocarcinoma (2/21; 9.5%), and carcinosarcoma (1/21; 4.8%).

In 1925, Sampson [5] proposed the criteria for the diagnosis of malignancy arising in endometriosis as follows: (1) demonstration of both benign and neoplastic endometrial tissues in the tumor, (2) the histology being compatible with endometrial origin, and (3) no other primary tumor sites being found. Further, in 1953, Scott [6] postulated a fourth criterion; that is, (4) the morphologic demonstration of benign endometriosis contiguous with the malignant tissue is a prerequisite for adjudication of a malignancy originating in endometriosis. In the present case, the first three criteria were fulfilled. Considering the fourth criterion in our patient, benign endometrium-like tissues were found adjacent to the clear cell carcinoma lesion (Figures 2(b) and 2(c)), suggesting that the majority of the endometriosis tissue in the abdominal wall had been replaced by cancer tissue.

Clear cell carcinoma is known as a representative histological type in renal cell cancer, pancreatic cancer, adrenal cancer, and gynecological cancer [7]. Immunohistochemical analysis has shown that tumor cells in gynecological cancer tissues stain positive for CK7 and negative for CK20 [7], while glypican-3 is generally positive in hepatocellular cancer and renal cell cancer. In this case, immunohistochemistry was positive/negative for CK7/CK20 and negative for glypican-3, suggesting that this tumor could be metastatic tissue from gynecological cancer. Based on this evidence, we diagnosed this tumor as malignant transformation from abdominal wall endometriosis after cesarean section.

In all the 21 cases found in the literature, surgical treatment was performed for malignant transformation of abdominal wall endometriosis. Among these, local resection was performed in 10 cases (47.6%), and total abdominal hysterectomy or salpingo-oophorectomy was conducted in 11 cases (52.4%). However, no malignant lesions were observed in either the uterus or both the adnexa that were resected, suggesting that the significance of the extensional resection, including hysterectomy or oophorectomy, remains unclear. For our patient, we chose only local resection of the lesion since PET-CT did not show the presence of any malignant lesions. However, the patient experienced a relapse lesion in the same area of the cesarean section scar at 8 months after the first surgery. Previous reports showed that 9 patients (42.9%) underwent relapse and 7 patients (33.3%) died of this disease. Once recurrence occurred, no treatment—including chemotherapy and radiation therapy—was effective. Most cases shown in Table 1 occurred subsequent to cesarean section. With the increasing incidence of cesarean sections, the number of cases with similar malignant transformation of abdominal wall endometriosis may be expected to increase. Further studies are necessary to determine the optimal treatment for malignant transformation of abdominal wall endometriosis.

## Figures and Tables

**Table 1:** Twenty-one cases of malignant transformation from abdominal wall endometriosis.

No.	Reference	Age (years)	Previous surgery	Delay (years)	Histology	Coexisting endometriosis on histology	Surgical treatment	CT	RT	Followup (months)	Outcome
1.	Schnieber and Wagner-Kolb [8]	40	CS	15	CCC	Yes	LR, TAH + BSO	−	+	18	DOD
2.	Hitti et al. [9]	46	CS	14	CCC	Yes	LR, TAH + BSO	−	−	30	NED
3.	Markopoulos et al. [10]	50	CS	25	EC	No	LR	−	−	24	NED
4.	Gücer et al. [11]	45	CS	8	EC	Unclear	LR	−	−	20	DOD
5.	Miller et al. [12]	38	CS	9	CCC	Yes	LR, TAH + BSO	−	+	60	NED
6.	Park et al. [13]	54	CS	26	CCC	Yes	LR	−	+	6 weeks	NED
7.	Ishida et al. [14]	56	CS	24	CCC	No	LR	−	+	48	DOD
8.	Matter et al. [15]	60	CS	41	EC	Yes	LR	−	−	18	NED
9.	Li et al. [16]	38	CS	10	SC	No	LR, TAH + BSO + OMT	−	−	14	NED
10.	Leng et al. [17]	41	CS	16	Carcinosarcoma	Yes	LR	−	−	15	DOD
11.	Sergent et al. [18]	45	CS	28	CCC	No	LR, BSO	+	−	6	DOD
12.	Harry et al. [19]	55	CS	30	CCC	Yes	LR	−	+	18	NED
13.	Bats et al. [20]	38	CS	13	CCC	Yes	LR, TAH + BSO	+	−	2	NED
14.	Williams et al. [21]	53	CS	17	CCC	No	LR, TAH + BSO	+	+	11	DOD
15.	Matsuo et al. [22]	37	LC	10	CCC	No	LR, TAH + BSO + OMT, PEN	+	−	18	REC
16.	Omranipour and Najafi [23]	59	D & C^*^	20	SC	No	LR	+	−	12	NED
17.	Bourdel et al. [24]	43	CS	Unclear	CCC	Unclear	LR, TAH + BSO	+	+	22	DOD
18.	Shalin et al. [7]	47	CS	Unclear	CCC	Yes	LR	+	+	7	NED
19.	Mert et al. [25]	42	CS, USO	Unclear	CCC	Yes	LR, TAH + BSO	+	−	1	NED
20.	Mert et al. [25]	51	CS, TAH	Unclear	CCC	Yes	LR, BSO + OMT	−	+	31	NED
21.	This case	60	CS	35	CCC	Yes	LR	−	−	8	REC

BSO: bilateral salpingo-oophorectomy, CCC: clear cell adenocarcinoma, CS: cesarean section, D & C: dilatation and curettage, DOD: died of disease, EC: endometrioid adenocarcinoma, LC: laparoscopic cystectomy, LR: local resection, NED: no evidence of disease, OMT: omentectomy, PEN: pelvic lymph nodes dissection, REC: recurrence, SC: serous adenocarcinoma, TAH: total abdominal hysterectomy, USO: unilateral salpingo-oophorectomy, and ^*^laparotomy for perforation of the uterus during D & C.
